# Midkine: A Novel Biomarker to Predict Malignancy in Patients with Nodular Thyroid Disease

**DOI:** 10.1155/2016/6035024

**Published:** 2016-06-30

**Authors:** Fatih Kuzu, Dilek Arpaci, Mustafa Unal, Ayfer Altas, Gürkan Haytaoglu, Murat Can, Figen Barut, Furuzan Kokturk, Sevil Uygun Ilikhan, Taner Bayraktaroglu

**Affiliations:** ^1^Division of Endocrinology and Metabolism, Department of Internal Medicine, Faculty of Medicine, Bulent Ecevit University, 67600 Zonguldak, Turkey; ^2^Department of Internal Medicine, Faculty of Medicine, Bulent Ecevit University, 67600 Zonguldak, Turkey; ^3^Department of Biochemistry, Faculty of Medicine, Bulent Ecevit University, 67600 Zonguldak, Turkey; ^4^Department of Pathology, Faculty of Medicine, Bulent Ecevit University, 67600 Zonguldak, Turkey; ^5^Department of Biostatistics, Faculty of Medicine, Bulent Ecevit University, 67600 Zonguldak, Turkey

## Abstract

*Background*. Midkine (MK), a new heparin-binding growth factor, plays important roles in a variety of biological phenomena such as carcinogenesis, inflammation, and angiogenesis. In this study, we aimed to evaluate serum midkine (SMK) and nodular midkine (NMK) levels in patients with thyroid nodules to predict malignancy and whether there was any association between.* Methods*. A total of 105 patients (74 women, 31 men) with thyroid nodules were enrolled. The levels of SMK and NMK were measured. Any possible correlation between SMK, NMK, and biochemical, cytopathological, or radiological variables was investigated.* Results*. Both SMK and NMK were found to be higher in hypoechoic nodules with an irregular border and without a halo (*p* < 0.05). Serum MK levels were significantly higher in nodules with microcalcifications than nodules with macrocalcification or without calcification (*p* = 0.001). SMK levels were found to be correlated with NMK levels (SMK 0.63 ng/ml versus 1.04 ng/mL and NMK 0.55 ng/mL versus 0.55 ng/mL, *r* ^2^ = 0.54, *p* < 0.001).* Conclusion*. Both SMK and NMK can predict tumorigenesis of highly malignant/suspicious thyroid cytopathology and also well correlated with sonographic features of thyroid nodules. We suggest that MK levels may serve as an alternative biomarker, in conjunction with the cytopathological results in preoperative assessment of thyroid nodules.

## 1. Introduction

Thyroid nodules are a common clinical problem. The prevalence of malignancy in thyroid nodules is currently about 5–15% [1]. Optimal prediction of malignancy in nodular thyroid disease is needed to achieve the best medical and surgical intervention. Fine needle aspiration biopsy (FNAB) is widely used and has improved preoperative prediction of malignancy but still has disadvantages including operator variability and nondiagnostic reports. Therefore researchers have focused on identifying novel biologic markers that might be associated with malignancy in thyroid nodules [1–4].

Midkine (MK), a novel heparin-binding growth factor, plays critical roles in a variety of biological phenomena such as carcinogenesis, inflammation/immunity, blood pressure, cellular proliferation, survival, migration of cellular functions, angiogenesis, fibrinolysis, and host defense and tissue protection [5–11]. The MK gene is located on human chromosome 11 [7]. Human MK is 13 kDa and contains 143 amino acids [8]. There was release of MK from cancer tissue into the blood. SMK was found in 87% percent of various cancers. High serum and tissue MK levels have been proposed as indicative of malignancy in numerous tumors and to be prognostic markers of their behavior [11–20]. However, in papillary thyroid cancer (PTC), tissue MK overexpression has been reported to be in correlation with clinicopathological features of the tumor, hypothesizing that MK might play a role as a biomarker for diagnosis and more aggressive behavior of papillary thyroid cancer such as lymph node metastasis and extrathyroidal invasion [4, 21]. Also they found that benign adenomatoid nodules showed less MK overexpression than the malignant nodules [4]. The studies for SMK in differentiated thyroid cancer were very rare. Meng et al. found that SMK level was higher in differentiated thyroid cancer than benign thyroid lesions [22]. Moreover, we hypothesized that higher levels of MK might be associated with malignant thyroid nodules. The confirmation of the hypothesis which was mentioned above might shed some valuable light on the evaluation of thyroid nodule within diagnostic and/or prognostic perspectives. From this standpoint, this prospective study was designed to evaluate the value of serum and nodular MK levels that is associated with tumorigenesis and nodulations.

## 2. Materials and Methods

### 2.1. Subjects

This prospective study included 105 subjects between the ages of 26–82 with nodular goiter admitted to the endocrinology department of a tertiary care center. This study was approved by the local Institutional Review Board and written informed consent was obtained from every patient included in this trial.

### 2.2. Blood Samples

Antecubital venous blood samples were taken in the morning after 12 h fasting for free triiodothyronine (FT3), free thyroxine (FT4), thyroid stimulating hormone (TSH), and sedimentation. Thyroid function tests (TSH, FT3, and FT4) were measured by direct chemiluminescence method (*Advia Centaur XP, Siemens, Dublin, Ireland*). Normal limits were as follows: FT3: 1.8 to 4.7 pg/mL, FT4: 0.8 to 2.6 pg/mL, and TSH: 0.4 to 6 *μ*IU/mL.

### 2.3. Imaging

Thyroid scintigraphy scans of the patients were done by giving pertechnetate (5 mCi FTc). Thyroid ultrasounds of patients enrolled in the study were performed by using a high-resolution ultrasound device with 7.5 MHz probe, prior to FNAB. Sonographic features of thyroidal nodules were evaluated and recorded: size with three dimensions, nodular structure (pure solid, cystic, and mixed), echogenicity (hypoechoic, isoechoic, and hyperechoic), nodular contour (smooth, irregular), presence of peripheral halo, and presence and type of calcification (microcalcification, macrocalcification).

### 2.4. Fine Needle Aspiration Biopsy

FNAB was performed with ultrasound-guided FNAB using a 22-gauge needle and 10cc syringe. Two samples were obtained from each nodule.

### 2.5. Serum and Aspirate Midkine

To obtain the sera of patients, venous blood samples were centrifuged for 5 minutes at 5000 rpm. Thyroid fine needle aspiration materials were centrifuged at 2000 rpm for 5 min. Midkine level (ng/mL) in serum and nodularity received from aspiration materials were studied by using commercially available OmniKine Human Midkine ELISA Kits (*Assay Biotechnology Company, CA, USA*). Test measuring range was 15.625 to 2000 pg/mL.

### 2.6. Cytopathology

The results of FNAC were divided into two groups as benign and suspicious/malignant.

### 2.7. Statistical Analysis

Statistical evaluation was carried out by using SPSS program version 18.0 (*SPSS Inc., Chicago, IL, USA*). Appropriacy of the normal distribution of quantitative variables was analyzed with Shapiro-Wilk test. Descriptive statistics for numeric variables were defined as mean ± standard deviation and median (minimum–maximum) and as numeric and percentage for the categorical data. Differences between the groups in terms of categorical variables were examined by chi-square test. Quantitative variables were compared in the two groups via the Mann-Whitney* U* test, while Kruskal-Wallis variance analysis was used for comparison of the three groups. Pairwise comparison of subgroups in the Kruskal-Wallis variance analysis was made by Bonferroni-corrected Mann-Whitney* U* test. The relationship between two numerical variables was examined using Spearman's correlation analysis. Results were evaluated in 95% confidence interval and *p* value < 0.05 was considered statistically significant.

## 3. Results

A total of 105 cases were enrolled in this study. Among these cases, 74 (70%) cases were women and the mean age was 51.9 ± 14.4 years. Median TSH, median SMK, and median NMK levels were 0.84 (0.004–64.3) *μ*IU/mL, 0.62 (0.30–4.97) ng/mL, and 0.53 (0.35–1.73) ng/mL, respectively. There was no statistically significant difference for both SMK and NMK based on gender, age, and TSH levels (*p*> 0.05) (Table 1).

Although the levels of SMK and NMK were found to be higher in the patients with hypoactive nodules than in the patients with iso/hyperactive nodules, no statistically significant difference was found (*p* = 0.44 and *p* = 0.119). We also could not identify significant differences between the groups according to nodule volumes for both SMK and NMK (*p* = 0.809 and *p* = 0.658, resp.) (Table 2).

Both SMK and NMK were found to be higher in hypoechoic nodules compared to iso/hyperechoic nodules; in irregular border compared to regular border; in nodules with absent or irregular halo compared to clear halo as shown in Table 2.

SMK levels were detected as being significantly higher in nodules containing microcalcifications than those with macrocalcification or without calcification (*p* = 0.001). There was no significant difference between the levels of MK for patients with nodules containing macrocalcification and no calcification. No statistically significant difference was detected in NMK levels between three groups (*p* = 0.308). Similarly, no differences were encountered with respect to SMK and NMK levels in terms of structure and the number of nodules (Table 2).

**Figure 1:**
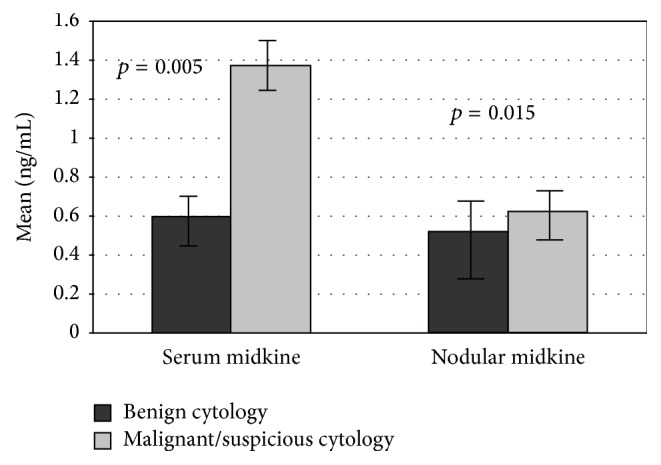
Serum and nodular MK levels evaluated according to FNA cytology.

According to cytopathology, 71 cases were in Group 1 and 34 in Group 2. Both SMK and NMK levels were higher in malignant/suspicious cytology group than benign cytology group (*p* = 0.005 and *p* = 0.015) (Table 3) (Figure 1).

When a cut-off level of 0.63 was considered for SMK among FNA results, *p* was found to be 0.001 for under or over 0.63 (AUC = 0.790). When a cut-off level of 0.57 was considered for NMK among FNA results, *p* was found to be 0.010 for under or over 0.57 (AUC = 0.750).

A total of 50 cases which were included in the study had undergone thyroid surgery (surgical indications were big nodule size, suspicious or malignant thyroid cytology, medical recurrence hyperthyroidism, and preferences of patients). The postoperative histopathological examination yielded follicular adenoma in 10 patients, nodular hyperplasia in 28 patients, papillary carcinomas in 10 patients, and follicular thyroid cancer in 2 cases.

**Figure 2:**
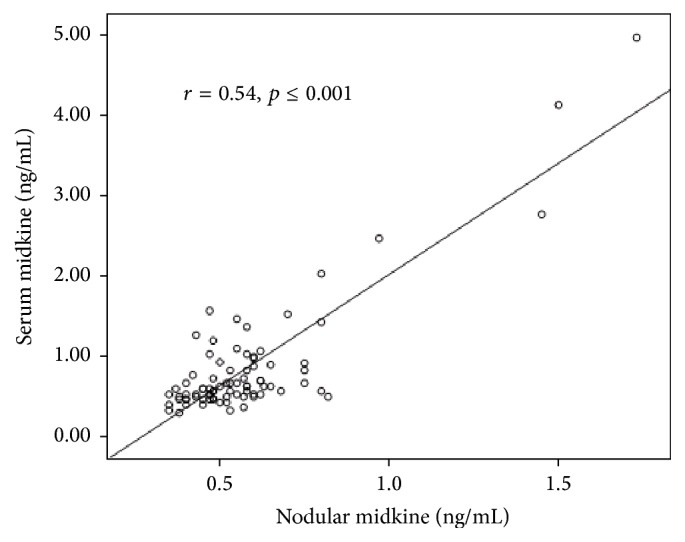
Correlation between serum and nodular midkine levels.

The levels of SMK and NMK were insignificantly higher in subjects with differentiated thyroid carcinoma than in the patients with follicular adenoma or nodular hyperplasia (*p* = 0.066 and *p* = 0.341, resp.) (Table 3). The levels of SMK correlated with NMK levels (*r* = 0.54, *p* < 0.001) (Figure 2).

## 4. Discussion

In the present study, the evaluation of SMK and NMK levels in patients with thyroid nodules in the probable association of MK levels and sonographic, cytological, and histopathological features of the thyroid nodules was targeted. We found that both SMK and NMK concentrations in thyroid nodules were significantly different in malignant nodule compared to benign nodule.

Diagnosis of thyroid nodules has been facilitated by popularization of high-resolution US and whenever thyroid nodules are discovered clinically or incidentally, exclusion of malignancy gains importance. Fine needle aspiration cytology is still the most reliable and the most accurate and cost-effective method for preoperative evaluations [1, 2]. However, its predictive value is still limited. Because it is invasive, the detection of malignancy depends in part on operator experience and may vary with respect to technical performance, nondiagnostic cytology rate is high, and also malignancy cannot be excluded in about 25% of thyroid nodules, possibly leading to unnecessary thyroid surgery [2, 3]. Due to this limitation, researches have focused on genetic (BRAF, RAS, and RET/PTC) and biological (galactine-3, HBME-1, and cytokeratin 19) markers that may aid in diagnosis and follow-up [1, 4, 23]. Midkine is a heparin-binding growth factor that plays roles in growth, survival, inflammation/immunity, blood pressure, cellular proliferation, migration of cellular functions, angiogenesis, fibrinolysis, host defense and tissue protection, neurogenesis, and carcinogenesis [9–11, 24–29]. It may enhance tumor invasion and therefore influence rates of survival [20–22].

In some precancerous lesions, SMK levels have been found to be increased [12, 18, 19]. Overall, MK expression is closely related with progression of tumor stage and poor prognosis such as neuroblastomas, glioblastomas, and bladder carcinomas [21]. If tumor tissues increase secretion of MK, MK becomes evident in serum. Some publications suggest that SMK levels have been increased in some precancerous lesion [12, 18, 19]. The expression of MK gene in human tumor cells may reflect tumor formation and give clues to the biological behavior of neoplasms. Hence, the expression of MK may serve as a tumor marker for diagnosis and follow-up [5]. From another point of view, blockade or knockdown of MK can constitute an effective option for cancer therapy [19]. A limited number of previous studies indicated that MK expression did not occur in normal thyroid tissue, but MK expression is mainly derived from the tumor tissue of PTC patients [4, 19]. In two different publications, MK expression was found to be correlated with aggressive clinicopathological features of PTC. They suggested that MK could be a reliable biomarker for diagnosis and prognosis of PTC [4, 21]. To the best of our knowledge, this is the second study that investigates both serum and nodular MK levels in thyroid nodules and malignancy. The first was performed by Jee et al. [30]. They found that higher MK concentrations in FNAB materials were obtained from PTC than the MK concentrations found in patients with benign thyroid disease.

Similar to their results, we found that both SMK and NMK levels were higher in malignancy/suspicious nodules compared with benign nodules. Also we found that SMK and NMK levels were higher among patients with suspicious ultrasound features for malignancy such as presence of microcalcification, irregular border, hypoechoic, hypoactive, and heterogeneous components, and absence of halo. Jee et al. also determined higher MK concentrations and lower Tg concentrations associated with higher MK/Tg ratio in PTC than the MK concentrations in benign nodular thyroid disease and suggested that this ratio may be a tool for making diagnostic distinction between malignant and benign thyroid disease. In terms of histopathologic results of the 50 patients operated on, SMK was found to be higher in malignant thyroid disease when compared with benign counterparts. Nevertheless these differences did not reach statistical significance.

Ikematsu et al. [20] have showed that SMK in patients with cancer was significantly higher than controls. However in our study, patients with both benign nodules and malignant nodules exhibited SMK levels higher than 0.5 ng/mL. The previous study found no difference between gender and age groups as well as any tumors stage and size and also demonstrated a decrease in SMK levels after surgery [4, 20]. Similar to these results, we did not come across any differences between SMK/NMK levels and also volume of nodules. Lack of data on alteration of MK levels is a major limitation of our study. A correlation between SMK and NMK levels was determined in our study. Nevertheless, NMK levels in malignant thyroid nodules were not proportionately elevated as SMK levels of the same patients. This discordance might be attributed to the lower number of malignant cases in the presented study. Thus, further studies with a larger number of cases are required to comment on this issue.

In accordance with our study, Ikematsu et al. [20] found higher SMK concentrations in various cancer types and reported a reduction in SMK concentrations after surgery. Similarly, Jee et al. [30] reported that metastatic PTC had more MK concentrations than those without metastasis and argued that MK may be beneficial both in the diagnosis and in the prognosis of malignant thyroid disease. No lymph node metastasis has been determined in our patients who had undergone surgery. Accordingly, we are not capable of commenting on the effect of MK concentration on invasion and prognosis of malignant thyroid disease. Angiogenic and fibrinolytic activities of MK may help to enhance the spread of cancer by creating an appropriate microenvironment [10, 11]. Hence, MK may yield a target molecule for antitumor drugs. Indeed, an oligonucleotide that blocks MK suppressed tumor formation in mice with rectal carcinoma and inhibited the angiogenesis in tumoral tissue [10]. Several studies have demonstrated that interference with MK activity yields promising experimental results in chemotherapy for various cancers [11, 19, 31–35].

One of the limitations of the study is that only 50 out of 105 patients underwent surgery and then pathology to have their diagnosis confirmed. Another limitation was that we used only two classifications of the Bethesda system (benign and suspicious malignant/malignant). The primary aim of this study was to include patients with thyroid nodules detecting cytopathology. This is a preliminary study for patients with thyroid nodules for whom undergoing surgery with suspicious/malignant FNA was decided and could guide large series of patients with suspicious/malignant FNA.

## 5. Conclusions

The results of the presented study demonstrate that both SMK and NMK might be the indicators of highly malignant/suspicious thyroid cytopathology, suggesting that midkine might serve as a novel biomarker in conjunction with the cytopathological results in preoperative assessment of thyroid nodules. To guide clinical practice, further prospective trials with larger numbers of patients and long term follow-up are warranted to evaluate the actual diagnostic, prognostic, and therapeutic potentials of SMK and NMK. The present study explored the usefulness of midkine as a biomarker in the differentiation between benign and malignant thyroid nodules in samples from serum and FNAC.

## Figures and Tables

**Table 1:** Demographic and clinical characteristics in relation to serum midkine (SMK) and nodular midkine (NMK) concentrations.

Demographic and clinical characteristics	SMK	NMK
Gender		
Female	0.60	0.52
Male	0.63 (0.30–4.97)	0.68 (0.35–1.73)
*p*	0.912	0.223
Age (years)		
<40	0.67 (0.37–4.97)	0.56 (0.38–1.73)
40–59	0.60 (0.30–2.77)	0.52 (0.35–1.45)
≥60	0.60 (0.40–2.03)	0.55 (0.37–0.82)
*p*	0.396	0.298
TSH (*µ*IU/mL)		
<0.4	0.57 (0.33–2.03)	0.52 (0.35–0.82)
0.4–4	0.63 (0.30–4.97)	0.53 (0.35–1.73)
>4	0.63 (0.50–1.37)	0.58 (0.43–0.62)
*p*	0.467	0.529

BMI: body mass index; TSH: thyroid stimulating hormone.

**Table 2:** Sonographic correlations of SMK/NMK concentrations.

Sonographic features	SMK	NMK
Nodule volume		
<10 mL	0.60 (0.30–4.97)	0.52 (0.37–1.73)
10–20 mL	0.63 (0.33–4.13)	0.57 (0.35–1.50)
>20 mL	0.63 (0.33–2.47)	0.55 (0.35–0.97)
*p*	0.809	0.658
Nodular echogenicity		
Hypoechoic	0.90 (0.47–4.97)	0.59 (0.35–1.73)
Hyperechoic	0.55 (0.40–0.93)	0.55 (0.40–0.68)
Isoechoic	0.53 (0.30–1.47)	0.48 (0.35–0.80)
*p*	<0.001^*∗*^	0.003^*∗*^
Calcification		
Microcalcification	1.20 (0.60–4.13)	0.60 (0.43–1.50)
Macrocalcification	0.63 (0.37–1.10)	0.56 (0.35–0.65)
No calcification	0.57 (0.30–4.97)	0.52 (0.35–1.73)
*p*	0.001^*∗*^	0.308
Border		
Regular	0.59 (0.30–4.97)	0.49 (0.35–1.73)
Irregular	0.90 (0.37–2.77)	0.60 (0.40–1.45)
*p*	0.015^*∗*^	0.001^*∗*^
Halo		
Present	0.57 (0.30–4.97)	0.50 (0.35–1.73)
Absent	0.70 (0.37–4.13)	0.58 (0.35–1.50)
*p*	0.003^*∗*^	0.019^*∗*^
Nodule structure		
Heterogeneous	0.67 (0.30–4.13)	0.57 (0.35–1.57)
Homogeneous	0.53 (0.40–4.97)	0.48 (0.38–1.73)
*p*	0.023^*∗*^	0.031^*∗*^
Number of nodules		
Solitary	0.70 (0.47–2.77)	0.57 (0.38–1.45)
Multiple	0.60 (0.30–4.97)	0.53 (0.35–1.73)
*p*	0.198	0.356

(Hint: *∗*: statistically significant.)

**Table 3:** The relationship between SMK/NMK concentrations and fine needle aspiration cytology/histopathology results.

	SMK (median) (min–max)	Mean ± SD	95% CI	NMK (median) (min–max)	Mean ± SD	95% CI
Cytopathology						
Benign (*n* = 71)	0.60 (0.30–4.97)	0.77 ± 0.65	0.62–0.93	0.52 (0.35–1.73)	0.55 ± 0.21	0.50–0.60
Suspicious/ malignant (*n* = 34)	1.37 (0.50–4.13)	1.47 ± 1.11	0.61–2.32	0.62 (0.43–1.50)	0.72 ± 0.31	0.48–0.96
*p*	0.005	<0.001		0.015	0.005	
Histopathology						
Follicular adenoma/ nodular hyperplasia (*n* = 38)	0.63 (0.30–4.13)	0.78 ± 0.71	0.50–1.06	0.55 (0.35–1.50)	0.55 ± 0.20	0.47–0.64
Differentiated thyroid cancer (*n* = 12)	1.04 (0.48–1.53)	1.03 ± 0.42	0.58–1.47	0.57 (0.48–0.80)	0.59 ± 0.13	0.46–0.73
*p*	0.066	<0.001		0.341	0.050	

## Abbreviations

MK: Midkine
SMK: Serum midkine
NMK: Nodular midkine
FNAB: Fine needle aspiration biopsy
PTC: Papillary thyroid cancer
WBC: White blood cells
FT3: Free triiodothyronine
FT4: Free thyroxine
TSH: Thyroid stimulating hormone
BMI: Body mass index
Tg: Thyroglobulin.
